# Computational Simulations Identified Marine-Derived Natural Bioactive Compounds as Replication Inhibitors of SARS-CoV-2

**DOI:** 10.3389/fmicb.2021.647295

**Published:** 2021-04-21

**Authors:** Vikas Kumar, Shraddha Parate, Sanghwa Yoon, Gihwan Lee, Keun Woo Lee

**Affiliations:** ^1^Division of Life Sciences, Department of Bio & Medical Big Data (BK4 Program), Research Institute of Natural Science, Gyeongsang National University, Jinju, South Korea; ^2^Division of Applied Life Science, Plant Molecular Biology and Biotechnology Research Center (PMBBRC), Gyeongsang National University (GNU), Jinju, South Korea

**Keywords:** COVID-19, 3CLcpsdummypro, PLcpsdummypro, RdRp, molecular dynamics simulations (MD), MM/PBSA binding free energy, pharmacokinetic properties

## Abstract

The rapid spread of COVID-19, caused by the novel severe acute respiratory syndrome coronavirus 2 (SARS-CoV-2), is a worldwide health emergency. Unfortunately, to date, a very small number of remedies have been to be found effective against SARS-CoV-2 infection. Therefore, further research is required to achieve a lasting solution against this deadly disease. Repurposing available drugs and evaluating natural product inhibitors against target proteins of SARS-CoV-2 could be an effective approach to accelerate drug discovery and development. With this strategy in mind, we derived Marine Natural Products (MNP)-based drug-like small molecules and evaluated them against three major target proteins of the SARS-CoV-2 virus replication cycle. A drug-like database from MNP library was generated using Lipinski’s rule of five and ADMET descriptors. A total of 2,033 compounds were obtained and were subsequently subjected to molecular docking with 3CL^pro^, PL^pro^, and RdRp. The docking analyses revealed that a total of 14 compounds displayed better docking scores than the reference compounds and have significant molecular interactions with the active site residues of SARS-CoV-2 virus targeted proteins. Furthermore, the stability of docking-derived complexes was analyzed using molecular dynamics simulations and binding free energy calculations. The analyses revealed two hit compounds against each targeted protein displaying stable behavior, binding affinity, and molecular interactions. Our investigation identified two hit compounds against each targeted proteins displaying stable behavior, higher binding affinity and key residual molecular interactions, with good *in silico* pharmacokinetic properties, therefore can be considered for further *in vitro* studies.

## Introduction

The world is experiencing a global public health emergency due to the outbreak of coronavirus disease 2019 (COVID-19), which is caused by a novel virus known as severe acute respiratory syndrome coronavirus-2 (SARS- CoV-2; Gorbalenya et al., 2020; Sohrabi et al., 2020). The outbreak was first identified in Wuhan, China (Huang et al., 2020; She et al., 2020; Zhu et al., 2020), but has since spread all around the world. As of December 1, 2020, more than 61.8 million confirmed cases of COVID-19, including 1.4 million deaths, have been reported to the WHO. According to the WHO and recent studies, the most common symptoms of SARS-CoV-2 infection are fever, a dry cough, and fatigue. The infection can also lead to pneumonia, SARS, and death in severe cases (Chen et al., 2020a; Wang et al., 2020a). The novel virus belongs to the Coronaviridae family, a type of positive-sense, single-stranded enveloped RNA virus. There are four classes of coronaviruses designated as alpha, beta, gamma, and delta. The betacoronavirus class includes SARS coronavirus (SARS-CoV), Middle East Respiratory Syndrome (MERS) coronavirus (MERS-CoV), and the COVID-19 causative agent SARS- CoV-2 (Pal et al., 2020). In the past, before the discovery of SARS-CoV-2, six types of coronaviruses were known to cause diseases in humans, including SARS-CoV and MERS-CoV (Mathpal et al., 2020; Zhu et al., 2020). The genome analysis of SARS-CoV-2 sequences revealed that it shares a 79% sequence identity with SARS-CoV and 89% with bat SARS-CoV (Lu et al., 2020). This implies that SARS-CoV-2 might have originated in bats (Boni et al., 2020). The genome of the virus is approximately 30 kB long, consisting of two large polyproteins; ORF1a and ORF1b (that proteolytically cleaves to form 16 nonstructural proteins), four structural proteins, and six accessory proteins (Snijder et al., 2003; Khailany et al., 2020; Yoshimoto, 2020). The structural proteins of SARS-CoV-2 are the enveloped protein (E protein) which helps in morphogenesis during the assemblage of the viral genome, the membrane protein (M protein) which maintains the shape of the virus envelope, and the nucleocapsid (N protein) which is responsible for RNA binding and dimerization (Fehr and Perlman, 2015; Schoeman and Fielding, 2019; Astuti and Ysrafil, 2020). The viral glycosylated spike (S) protein is responsible for the entry of both SARS-CoV and SARS-CoV-2 into the host *via* interacting with a receptor protein called angiotensin-converting enzyme 2 (ACE2), found on the surface membrane of the host cells (Tai et al., 2020). After the entry of the SARS-CoV-2 into the cell, the replication of the coronavirus starts with the translation of two overlapping open reading frames (ORF1a and ORF1b) to produce pp1a and pp1ab, generated by a ribosomal frameshift mechanism (Astuti and Ysrafil, 2020; Gul et al., 2020; Kim et al., 2020). These polyproteins are then processed proteolytically by two essential viral cysteine proteases-papain-like protease or PL^pro^ (non-structural protein 3 or nsp 3) and 3C-like protease (3CL^pro^, also known as the main protease, M^pro^, or nsp5) (Chen et al., 2020b). Cleavage of pp1a and pp1ab results in the production of 16 non structural protein (NSP)s, with PL^pro^ and 3CL^pro^ (Prajapat et al., 2020). Nsp12 is the first ORF1b encoded protein and has viral RNA-dependent RNA polymerase (RdRp) activity, it has been reported that it requires nsp7 and nsp8 co-factors for maximal activity of its replication and transcription of positive and negative strands of RNA (Kirchdoerfer and Ward, 2019; Hillen et al., 2020). The role of other NSPs were also reported to play specific functions in viral replication and transcription and has been reviewed in detail (Astuti and Ysrafil, 2020; Chen et al., 2020b). Since the outbreak, researchers have repurposed several drugs that could have potential effectiveness against COVID-19. Currently there are no clinically proven specific antiviral agents available for SARS-CoV-2 infection and researchers have been battling to discover promising cures to save lives. Recently, researchers have targeted viral structural and non-structural proteins using computational drug repurposing as well as experimental approaches (Beck et al., 2020; Caly et al., 2020; Chang et al., 2020; Elfiky and Ibrahim, 2020; Gao et al., 2020; Jin et al., 2020; Li and De Clercq, 2020; Li et al., 2020; Ma et al., 2020; Sekhar, 2020; Ton et al., 2020; Wang et al., 2020b). Identified drugs were not able to treat infections to a significant extent and further research is therefore required for effective drugs or vaccine candidates against SARS-CoV-2. It has been reported that drugs like Lopinavir, Ritonavir, Remdesivir, Umifenovir, and Favipiravir are under clinical trials against SARS- CoV-2 target proteins (Drożdżal et al., 2020; Pawar, 2020), however, these drugs have been reported to show severe side effects in COVID-19 patients (Fan et al., 2020a; Khan et al., 2020b; Türsen et al., 2020). Simultaneously, researchers have focused on the identification of natural compounds *via* computational as well as experimental methods for SARS-CoV-2 inhibition and have identified potent molecules (Alagu Lakshmi et al., 2020; Alexpandi et al., 2020; Antonio et al., 2020; Bhuiyan et al., 2020; Chikhale et al., 2020; Pandey et al., 2020; Wang and Yang, 2020; Kumar Verma et al., 2021). Recently published reports summarized the role of natural compounds and revealed that a few of them are under clinical trials against COVID-19 (Khalifa et al., 2020; Kumari et al., 2020). Alternative sources of natural compounds against SARS-CoV-2 are of marine origin, and have not been explored in great detail and this can serve as an invaluable approach in the ongoing search for novel COVID-19 inhibitors. The marine environment is a rich source of chemically varied, biologically active natural compounds. Marine chemical compounds include polyketides, terpenes, nitrogen containing compounds, and polysaccharides (Uzair et al., 2011). It has been well-documented that marine compounds show antibacterial, antifungal, anti-malarial, and antiviral activities (Choudhary et al., 2017; Riccio et al., 2020). Antiviral activity of marine compounds has been tested against human immunodeficiency virus-1 (HIV-1), herpes simplex virus-2 (HSV-2), polio virus, dengue virus, measles virus, influenza virus, and SARS virus (Yasuhara-Bell and Lu, 2010; Uzair et al., 2011; Gogineni et al., 2015). A polyphenol from marine algae, namely dieckol, was described to be the most active agent against SARS-CoV M^pro^ through *in vitro* studies (Park et al., 2013). Similarly, coumarin derivatives from a marine sponge were also observed to display *in vitro* activities against SARS-CoV M^pro^ (De Lira et al., 2007). In the same way, molecular docking-based studies recently identified potential leads of marine origin against SARS-CoV-2 M^pro^ (Gentile et al., 2020; Khan et al., 2020a; Vijayaraj et al., 2020). This literature survey and the recent studies on marine compounds have therefore inspired us to intensively search for additional potent inhibitors. We therefore designed a virtual screening study for the search of novel drug-like molecules against NSPs 3CL^pro^, PL^pro^, and RdRp—deemed as key proteins required for the virus replication cycle of SARS-CoV-2—hence considered as attractive drug targets (Chellapandi and Saranya, 2020; Li and De Clercq, 2020; Muhammed, 2020).

## Materials and Methods

### Ligand Preparation

The Marine Natural Product (MNP) library was downloaded from Prof. Encinar’s website.^1^ Compounds were filtered for their drug-likeness properties such as the number of hydrogen bond donors, hydrogen bond acceptors, and AlogP values using Lipinski’s rule of five (Lipinski et al., 2001) and then for absorption, distribution, metabolism, and excretion properties using *ADMET descriptors* protocol in Discovery Studio (DS) v18 (www.accelrys.com Accelrys Inc. San Diego, CA, United States). The filtered compounds were energy minimized using the steepest descent algorithm with CHARMm forcefield implemented in DS. The optimized drug-like compounds were saved in Sybyl Mol 2 format for further processing of molecular docking.

### Preparation of Protein Structures

The crystal structure of the selected proteins 3CL^pro^ (PDB ID: 6Y2F) (Zhang et al., 2020), PL^pro^ (PDB ID: 6WX4) (Rut et al., 2020) and RdRp (PDB ID: 6M71) (Gao et al., 2020) were downloaded from RCSB Protein Data Bank (PDB).^2^ The 3CL^pro^ was in complex with its substrate α-ketoamide inhibitor 13b, which was reported to inhibit 3CL^pro^ with IC50 0.67 μM, while PL^pro^ was complexed with a peptide inhibitor VIR251, which was identified through a substrate hydrolysis assay. Additionally, two anti-retroviral drugs, Lopinavir and Ritonavir, which were found effective against SARS-CoV-2 proteases in *in silico* and *in vitro* studies were considered for comparison with our results against 3CL^pro^ and PL^pro^ (Fan et al., 2020b; Joshi et al., 2020; Mohamed et al., 2021; Servidio and Stellacci, 2021). During the course of this study, the crystal structure of RdRp complexed with its inhibitor substrate was unavailable. However, as per recent reports, Remdesivir was identified as a potent inhibitor of RdRp with EC50 0.77 μM (Wang et al., 2020b). Therefore, Remdesivir was considered as a reference compound against RdRp in our study. The three protein structures were then prepared in DS using *Clean Protein* tool, water molecules were removed, and hydrogens were added. Protein structures were then energy minimized with *Steepest Descent* algorithm in DS.

### Molecular Docking

The prepared protein structures (3CL^pro^, PL^pro^, and RdRp) were subjected to molecular docking with energy minimized drug-like molecules generated from the MNP library. The bound inhibitors of 3CL^pro^ and PL^pro^ were used to define the active site for docking, whereas the catalytic site for RdRp was defined by considering Remdesivir binding residues in DS (Gao et al., 2020). The docking calculation was performed using *Genetic Optimization of Ligand Docking* (GOLD v5.2.2) (Jones et al., 1997). The Goldscores (high) and Chemscores (low) of drug-like molecules were used for scoring and rescoring, respectively (Kumar et al., 2017, 2019). The Goldscore is used as a major scoring function as it measures the binding affinity of the ligand, whereas Chemscore calculates the total free energy change when a ligand binds to the protein (Verdonk et al., 2003; Bavi et al., 2017). Ten poses were generated for each drug-like ligand and the optimal pose was selected on the basis of scoring functions and key intermolecular interactions with active site residues of the protein.

### Molecular Dynamics Simulations

Selected compounds were further subjected to molecular dynamics (MD) simulations using *Groningen Machine for Chemical Simulations* (GROMACS v5.1.5) under physiological conditions (Pronk et al., 2013). The parameters and coordinate files for 3CL^pro^, PL^pro^, RdRp, and selected potential hit compounds were generated by CHARMMm27 forcefield (Sapay and Tieleman, 2011) in GROMACS and SwissParam (Zoete et al., 2011), respectively. The TIP3P water model was used for each simulation system which was neutralized by addition of 2Na^+^ ions (3CL^pro^), 1Cl^–^ ion (PL^pro^), and 12 Na^+^ ions (RdRp) in a dodecahedron periodic box. Energy minimization was performed for 50,000 nsteps using the steepest descent algorithm to avoid steric clashes. Equilibration of each system was performed in two stages; the first phase was carried out under a constant number of particles, volume, and temperature (NVT) ensemble for 500 ps at 300 K, using the V-rescale thermostat (Bussi et al., 2007) and in the second phase, the pressure of each system was equilibrated for 500 ps at a constant number of particles, pressure, and temperature (NPT) at 1 bar using a Parrinello–Rahman barostat (Parrinello and Rahman, 1981). Each equilibrated system was simulated for 30 ns under periodic boundary conditions to avoid edge effects. During simulation, the LINCS algorithm (Hess et al., 1997) was used to restrain the bond of heavy atoms and Particle Mesh Ewald (PME) was used for electrostatic interactions (Darden et al., 1993). The MD trajectories were analyzed using DS, GROMACS, and Visual Molecular Dynamics (VMD; Hess et al., 1997).

### Binding Free Energy Calculations

Prediction of the binding affinities of small-molecule inhibitors with their biological targets plays an important role in prioritizing compounds to be evaluated experimentally (Rifai et al., 2019). In this study, we have exploited Molecular Mechanics Poisson-Boltzmann Surface Area (MM/PBSA) to predict the protein-ligand binding free energies (BFE), by implementing a GROMACS compatible tool “g_mmpbsa” (Kumari et al., 2014). To calculate the BFE, 40 snapshots of protein–ligand complexes were selected evenly from the last 10ns of the MD trajectories. The detailed methodology of the BFE calculation was followed as described in previous reports (Bavi et al., 2017; Kumar et al., 2019; Zeb et al., 2019). The protein–ligand BFE ΔGbind is calculated as:

$$\Delta \,G_{b\,i\,n\,d}=G_{c\,o\,m\,p\,l\,e\,x}-\left(G_{p\,r\,o\,t\,e\,i\,n}+G_{l\,i\,g\,a\,n\,d}\right)$$

The final Δ*G*_*bind*_ values for protein–ligand complexes were the average values from the 20 to 30 ns of the MD simulation trajectories.

### Evaluation of Pharmacokinetic Properties

Investigation of key pharmacokinetic properties (PK) of the final compounds is crucial before considering the identified compounds for further drug development (van de Waterbeemd and Gifford, 2003). Therefore, pharmacokinetic properties such as the absorption, distribution, metabolism, excretion, and toxicity (ADMET) of the final compounds were predicted by a machine-learning platform pkCSM^3^ (Pires et al., 2015). This computational information could affect the decision to begin with synthesis either *via* a traditional medicinal chemistry or *via* a combinatorial chemistry strategy (van de Waterbeemd and Gifford, 2003).

## Results

**FIGURE 1:**
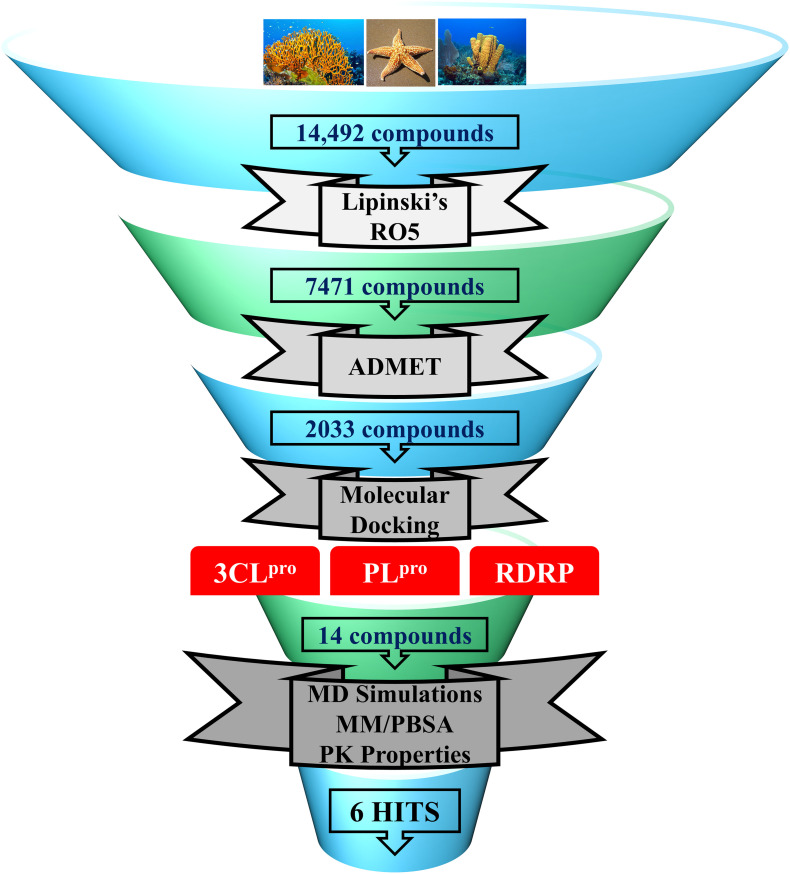
Schematic representation of methodologies used in the current study for the identification of potential COVID-19 replication inhibitors.

In the present study, a series of computational methods have been applied for the identification of potential compounds against COVID-19 replication proteins. The detailed schematic representation of the approach is shown in Figure 1.

### Selection of Marine Compounds

In the past, various marine natural compounds have been reported to display antibacterial, antifungal, anti- malarial, and antiviral activities (Choudhary et al., 2017; Riccio et al., 2020). Therefore, in the present study, the MNP library of 14,492 compounds was utilized for virtual screening against SARS-CoV-2 replication proteins. A drug-like database was generated as previously mentioned, and the compounds were then minimized for further screening. A total of 2,033 marine drug-like compounds were selected for further processing of molecular docking against three important SARS-CoV-2 replication proteins (3CL^pro^, PL^pro^, and RdRp). The three proteins were prepared by removing all the heteroatoms, adding hydrogens, and consequently minimized prior to docking.

### Molecular Docking

Molecular docking studies were performed to study the initial binding interactions of ligands with proteins using the GOLD program (Jones et al., 1997). The docking parameters were validated using REF inhibitors and the pose showing acceptable RMSD <2 Å for respective proteins were selected (Supplementary Figure 1). Ten conformers were generated for each drug-like compound and were subsequently analyzed on the basis of high Goldscore, low Chemscore, and key intermolecular interactions with target proteins (Kumar et al., 2017). The three-dimensional crystal structure of 3CL^pro^ with PDB ID: 6Y2F was used for molecular docking studies. The active site docking sphere was defined using the co-crystallized α- ketoamide inhibitor as a reference (REF) compound with a radius of 10.18 Å. The molecular docking analysis revealed that the REF compound displayed a Goldscore of 61.23 and a Chemscore of −17.76, and therefore, these scores were used as cut-off criteria to filter the drug-like compounds. Accordingly, 12 compounds exhibited a higher Goldscore and lower Chemscore than the REF compound, while only five of them demonstrated desirable hydrogen bond interactions with either one or more key residues of 3CL^pro^ reported earlier; –His41, Cys145, and Glu166 (Supplementary Table 1; Iftikhar et al., 2020; Wang, 2020; Zhang et al., 2020; Kumar et al., 2021). The molecular docking study for SARS-CoV-2 PL^pro^ was performed with PDB ID: 6WX4. The co-crystallized bound peptide inhibitor VIR251 was used as a REF for defining the active site with a 12 Å radius. The docking analyses revealed that the REF compound displayed a Goldscore of 56.09 and a Chemscore of -23.41. Accordingly, 10 compounds exhibited a higher Goldscore and lower Chemscore than the REF compound, while only four of them demonstrated hydrogen bond interactions with either of the key residues; Cys111, Asp164, Gly271, and Tyr278 (Supplementary Table 1; Naidoo et al., 2020; Rut et al., 2020; Shin et al., 2020). Molecular docking analyses revealed that both the drugs Lopinavir and Ritonavir displayed a lower docking score than the selected potential compounds against 3CL^pro^ and PL^pro^ (Table 1). In case of RdRp, the crystal structure with PDB ID: 6M71 was used as a receptor for the molecular docking study. The catalytic site was defined with a 13 Å radius using the only known inhibitor, Remdesivir, as a REF. The REF compound displayed a Goldscore of 60.95 and a Chemscore of -22.41. Correspondingly, 14 compounds were observed to display better scores than REF, while five compounds demonstrated favorable interactions with key residues Arg553, Arg555, Asp618, Asp760, Asp761, and Arg836 which are deemed essential for inhibition (Alexpandi et al., 2020; Elfiky, 2020; Iftikhar et al., 2020; Koulgi et al., 2020; Yin et al., 2020). The 2D structures, Goldscore, and Chemscore values of the selected compounds are shown (Supplementary Table 1).

**TABLE 1:** Molecular docking and molecular dynamics simulation analyses for potential hits.

Protein Name	Ligands	Docking scores		MD analyses	
		Goldscore	Chemscore	RMSD (backbone)	Binding free energy (ΔG) (kJ/mol)
3CL^pro^	Hit1	64.40	-29.37	0.16	–104.37
	Hit2	62.85	-28.33	0.15	–102.90
	REF	61.23	-17.76	0.13	–96.64
	Lopinavir	59.21	-22.20	0.16	–101.13
	Ritonavir	57.10	-19.56	0.14	–97.40
PL^pro^	Hit1	65.97	-31.82	0.17	–92.57
	Hit2	60.34	-32.20	0.18	–82.17
	REF	56.09	-23.41	0.16	–51.82
	Lopinavir	48.76	-30.05	0.19	–60.84
	Ritonavir	48.86	-21.11	0.21	–70.74
RdRp	Hit1	68.37	-25.65	0.29	–111.52
	Hit2	69.31	-22.65	0.26	–63.12
	Remdesivir	60.95	-22.41	0.29	–55.50

### Molecular Dynamics Simulation Analysis

Molecular dynamics simulation studies were performed to understand the stability, binding mode, and molecular interactions of potential compounds selected from molecular docking (Supplementary Table 1). A 30ns production run was performed using the GROMACS program. MD simulation analyses was performed for important properties like root mean square deviation (RMSD), BFE and identification of binding mode interaction. Only those compounds which displayed stable behavior throughout the simulation, the interaction with key residues, and the better binding affinity in terms of BFE than REF inhibitor, were considered for detailed analysis. The average structure was calculated from the last 10 ns of the simulation run for each protein–ligand complex to scrutinize the binding mode of the interaction of selected hit compounds with its respective protein structures. The compounds were further ranked according to BFE, calculated using the MM/PBSA tool (Table 1).

### Analysis of Stability and Binding Free Energy

**FIGURE 2:**
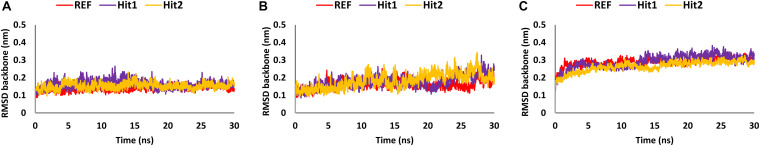
MD simulation analyses displaying backbone RMSD **(A)** 3CL^pro^, **(B)** PL^pro^, and **(C)** RdRp.

**FIGURE 3:**
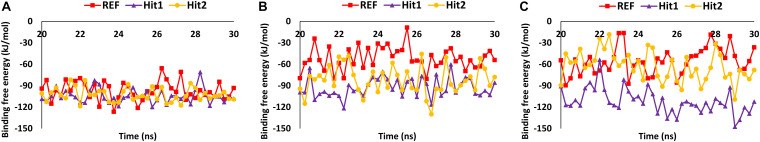
MM/PBSA binding free energy calculations. **(A)** 3CL^pro^, **(B)** PL^pro^, and **(C)** RdRp.

The stability of the simulated complexes was analyzed through backbone RMSD analysis and the BFE calculations. The RMSD analyses of Hit1, Hit2, and REF inhibitors for 3CL^pro^, PL^pro^, and RdRp (Figure 2) demonstrated that all hits remained stable throughout the 30ns of the production run and displayed convergent RMSD below 0.3 nm. Our results also confirms that Lopinavir and Ritonavir showed the previously mentioned behavior against 3CL^pro^ and PL^pro^ during the MD-simulation run, confirming that the Hit compounds can interact with the target proteins under simulated conditions (Supplementary Figures 2A,B). Simultaneously, binding free energies of the hits were calculated using MM/PBSA methodology. For this purpose, 40 frames were generated from the last 10 ns of trajectories displaying stable RMSD. The binding energies obtained were averaged and displayed (Table 1). The SARS-CoV-2 3CL^pro^ bound REF inhibitor α-ketoamide displayed a BFE of −96.85 kJ/mol, whereas Hit1 and Hit2 exhibited −104.37 and −102.90 kJ/mol of BFE, respectively. This analysis revealed that our hits have greater binding affinity towards 3CL^pro^ and can be considered for further evaluation (Table 1 and Figure 3A). Interestingly, the binding affinity of Lopinavir and Ritonavir for 3CL^pro^ was also found to be less than the selected Hits but the binding affinity of Lopinavir and Ritonavir was found to be better than the co-crystallized ligand 13b, used as REF in the study (Table 1 and Supplementary Figure 2C). In case of SARS-CoV-2 PL^pro^, the REF inhibitor VIR251 was found to display a BFE value of -51.82 kJ/mol, whereas Hit1 and Hit2 displayed a lower BFE value of -92.57 and -82.17 kJ/mol, respectively (Table 1 and Figure 3B). The binding affinity of Lopinavir and Ritonavir for PL^pro^ was found to be less than the selected Hits but interestingly, both drugs displayed better binding affinity than the Co-crystal inhibitor VIR251 (Table 1 and Supplementary Figure 2D). Similarly, SARS-CoV-2 RdRp REF inhibitor Remdesivir displayed a BFE value of -55.50 kJ/mol whereas, Hit1 and Hit2 was observed to demonstrate a BFE value of -111.52 and -63.12 kJ/mol, respectively (Table 1 and Figure 3C).

### Binding Mode and Intermolecular Interactions

**FIGURE 4:**
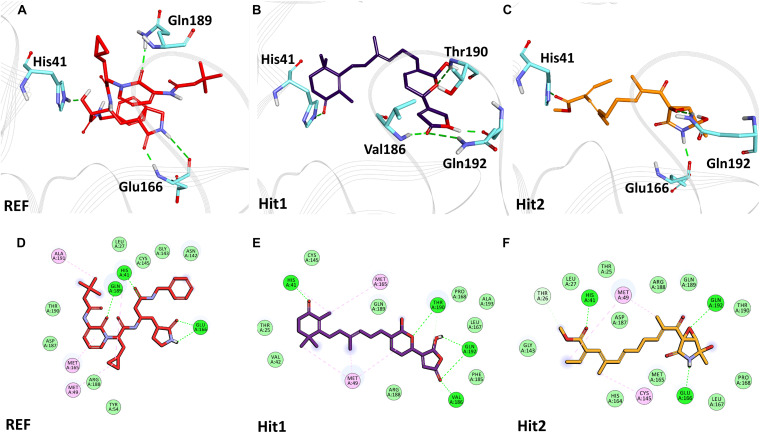
Binding mode of interaction in 3D (upper panel) for hits with the active site of SARS-CoV-2 3CL^pro^, **(A)** REF inhibitor, **(B)** Hit1 and **(C)** Hit2. The protein in background is shown as wire representation with gray color, interacting residues are displayed as cyan sticks while REF, Hit1 and Hit2 are displayed as red, purple and orange color sticks, respectively. The lower panel displaying 2D interactions, **(D)** REF, **(E)** Hit1 and **(F)** Hit2 with active site interacting residues of SARS-CoV-2 3CL^pro^. The hydrogen bonds are shown as green dashed lines while the π-alkyl and van der Waals interactions are displayed as pink and light green spheres, respectively.

**FIGURE 5:**
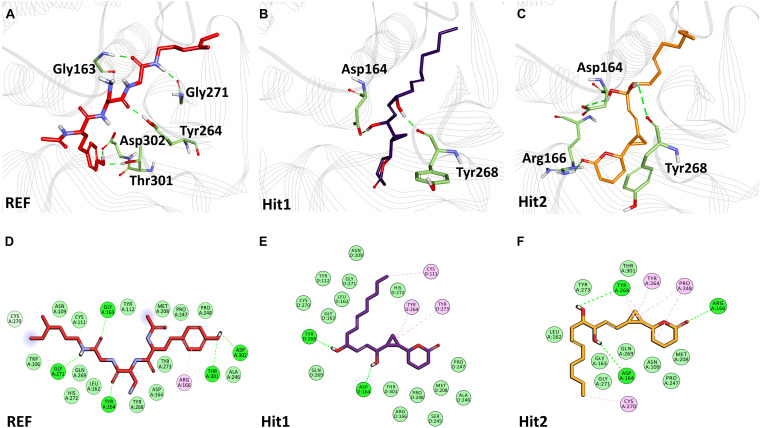
Binding mode of interaction in 3D (upper panel) for hits with the active site of SARS-CoV-2 PL^pro^, **(A)** REF inhibitor, **(B)** Hit1 and **(C)** Hit2. The protein in background is shown as wire representation with gray color, interacting residues are displayed as green sticks while REF, Hit1 and Hit2 are displayed as red, purple and orange color sticks, respectively. The lower panel displaying 2D interactions, **(D)** REF, **(E)** Hit1 and **(F)** Hit2 with active site interacting residues of SARS-CoV-2 PL^pro^. The hydrogen bonds are shown as green dashed lines, while the π-alkyl and van der Waals interactions are displayed as pink and light green spheres, respectively.

The binding mode of interaction for hits was analyzed by calculating the average structure from the last 10ns simulation trajectories. SARS-CoV-2 3CL^pro^ bound α-ketoamide REF inhibitor was observed to display three hydrogen bonds with catalytic site residues His41, Glu166, and Gln189. On the other hand, Hit1 formed four hydrogen bonds with catalytic site residues His41, Val186, Thr190, and Gln192, while Hit2 displayed three hydrogen bonds with residues His41, Glu166, and Gln192. The hit compounds were observed to demonstrate a significant number of non-covalent interactions including hydrogen bonds, van der Waals, and π-alkyl bonds with 3CL^pro^ (Figure 4 and Supplementary Table 2). Most significantly, our analysis revealed that both the hit compounds were found to target the catalytic triad (His41, Cys145, and Glu166) of 3CL^pro^ through hydrogen bonds or hydrophobic interactions. The binding mode of Lopinavir and Ritonavir for 3CL^pro^ was also analyzed and it was observed that both drugs target the catalytic triad residues, but the total number of hydrogen bonds observed are less than the Hit compounds (Supplementary Figure 3). The average structure of SARS-CoV-2 PL^pro^ bound REF inhibitor VIR251 was calculated, and the analysis revealed that the REF inhibitor was observed to demonstrate hydrogen bond interactions with Gly163, Gly271, Tyr264, Thr301, and Asp302. The identified Hit1 formed hydrogen bonds with key residues Asp164 and Tyr268 whereas, Hit2 exhibited hydrogen bonds with Asp164, Arg166, and Tyr268 (Figure 5 and Supplementary Table 2). It is worth noting that our hit compounds were found to target catalytically essential residues (Asp164 and Tyr268) of PL^pro^ through hydrogen bonds. The binding mode of Lopinavir and Ritonavir for PL^pro^ revealed that both drugs target the previously mentioned key residues (Supplementary Figure 4).With respect to RdRp, it was observed that the REF inhibitor Remdesivir, formed hydrogen bonds with Arg555 and Arg836 of RdRp. The binding mode of Hit1 was observed to be slightly different than the REF, and was found to target key residues Arg553, Arg555, Asp623, and Ala762 of the RdRp catalytic pocket. Moreover, Hit1 demonstrated significantly better binding affinity in terms of BFE than the REF inhibitor and was therefore considered as a potential hit (Table 1). The binding mode of Hit2 was observed to be similar to the REF and formed hydrogen bonds with key residues Ser549, Ala550, Arg555, and Asp761 (Figure 5 and Supplementary Table 2).

The overall MD simulation analyses revealed that our identified hits displayed stabilities throughout the production run, and demonstrated better binding affinities in terms of their energies with their respective protein partners and interactions with key residues of the active site. We therefore propose that our hits can be considered fit for further analysis against COVID-19.

### *In silico* Prediction of Pharmacokinetic Properties

The competence of therapeutic drugs mainly depends on their pharmacokinetic (PK) properties once within the system. The major parameters considered for prediction of these PK properties against 3CL^pro^, PL^pro^, and RdRp were calculated using an online machine-learning platform pkCSM (Table 2). Canonical SMILES IDs of ligands were generated using BIOVIA Draw and were then used as input in pkCSM. The server predicts absorption property of compounds considering Caco-2 permeability, which is widely used as an *in vitro* model to check the absorption level of orally administered drugs. The compounds showing values greater than 0.90 display a high Caco-2 permeability. Our hits presented with better Caco-2 permeability properties than REF compounds. Hits identified against 3CL^pro^ and PL^pro^ can easily cross the Caco-2 cell lines, whereas hits against RdRp displayed properties similar to the REF inhibitor Remdesivir, exhibiting less potential to cross the cell lines. Intestinal absorption (IA) below 30% indicates poor absorbance. The six identified hits displayed better IA properties than REF compounds. Interestingly, REF compound against PL^pro^ displayed poor IA. The blood brain barrier (BBB) acts as an extra border amidst the circulating blood and the extracellular space of the brain. It has been endorsed that the standard value to cross the BBB is >0.3 high to <-1 low logBB. The detailed BBB permeability (BBBP) analysis revealed that potential hits against three proteins cannot easily cross BBB, thereby demonstrating a lower chance of brain associated toxicity. Cytochrome P450s is considered as an essential enzyme for the metabolism of drugs in the liver. The two main subtypes of cytochrome P450 are CYP2D6 and CYP3A4. The inhibitor of these two enzymes can be metabolized in liver and henceforth, cannot be considered as an idyllic drug-like compound. Metabolism analysis revealed that the six identified hits against SARS-CoV-2 3CL^pro^, PL^pro^, and RdRp were not involved in CYP2D6 inhibition. Importantly, the hits against SARS-CoV-2 3CL^pro^ and PL^pro^ were not found to be CYP3A4 inhibitors, whereas Hit1 of RdRp including the REF compound can lead to its inhibition, as they were predicted to be CYP3A4 substrate inhibitors. The total clearance (TC) parameter helps to determine the dosing rate, indicating the rate of drug elimination by the body. The drug approaching the zero value is indicative of a lower clearance rate. The six identified hits were found to have a good TC property than REF compounds. As a final criterion, it is essential to test the toxicity of identified hits as this plays a pivotal role in further drug selection. In the present study, three important toxicity parameters namely AMES mutagenicity, hepatotoxicity (HP Tox), and skin sanitization (SS) were considered and studied in detail. It was observed that Hit2 of both 3CL^pro^ and PL^pro^ may be mutagenic, while other hits were demonstrated to be non-mutagenic. With respect to HP Tox PK property, Hit1 of RdRp and Hit2 of both 3CL^pro^ and PL^pro^ displayed a toxicity similar to the REF compounds of the three proteins, whereas other hits were observed to be non-toxic. Another parameter, namely SS, to test the toxicity of our hits was analyzed and our hits were perceived to be non-sensitive to the skin. From the above overall analyses, we can conclude that Hit1 of 3CL^pro^, Hit1 of PL^pro^, and Hit2 of RdRp have excellent *in silico* PK properties among our tested hits, whereas the remaining hits also display comparable properties when compared with standard values set by pkCSM and values of REF inhibitors.

**TABLE 2:** The pharmacokinetic properties for hits predicted by machine-learning platform pkCSM.

ADMET properties		3CL^pro^			PL^pro^			RdRp		
		Hit1	Hit2	REF	Hit1	Hit2	REF	Hit1	Hit2	REF
Absorption	Caco-2 permeability (log cm/s)	0.82	0.92	0.49	0.91	0.82	0.38	0.60	0.64	0.61
	IA human (% abs)	76.63	61.14	63.09	94.99	93.21	30.81	64.95	72.94	63.93
Distribution	BBBP (logBB)	-0.03	-0.44	-1.13	-0.39	-0.39	-1.41	-1.29	-0.87	-1.99
Metabolism	CYP2D6 substrate	No	No	No	No	No	No	No	No	No
	CYP2D6 inhibitor	No	No	No	No	No	No	No	No	No
	CYP3A4 substrate	No	No	Yes	No	No	No	Yes	No	Yes
	CYP3A4 inhibitor	No	No	No	No	No	No	No	No	No
Excretion	TC (ml/min/kg)	0.85	1.51	0.22	1.57	1.57	0.86	0.25	0.80	0.17
Toxicity	AMES toxicity	No	Yes	No	No	Yes	No	No	No	No
	HP Tox	No	Yes	Yes	No	Yes	Yes	Yes	No	Yes
	SS	No	No	No	No	No	No	No	No	No

*IA, intestinal absorption; BBBP, blood brain barrier permeability; TC, total clearance; HP Tox, Hepatotoxicity; SS, skin sensitization.*

## Novelty and Source Identification

As a final assessment, IUPAC names of our hits were generated using BIOVIA draw, and their chemical name and source of origin was identified (Table 3). Identified hits against 3CL^pro^ were found to originate from sponge and fungus, while PL^pro^ hits originated from red algae, and the RdRp identified hits were found to originate from sponge and starfish (John and Murray, 2008). Furthermore, the PubChem search engine^4^ was used for the purpose of a novelty search for our identified marine hits and it was found that these compounds were not reported against SARS-CoV-2 in any *in silico* and *in vitro* studies. Our identified hits can therefore be recommended as novel therapeutic SARS-CoV-2 replication inhibitors.

**TABLE 3:** Molecular structures and properties of identified hits against SARS-CoV-2 3CL^pro^, PL^pro^, and RdRp.

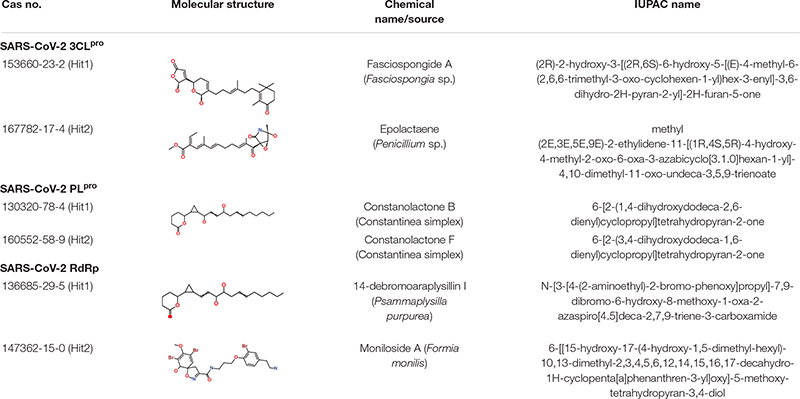

## Discussion

Identification of inhibitors that can bind and inhibit the proteins involved in viral replication has been the most effective strategy in antiviral drug discovery (Magden et al., 2005; Iftikhar et al., 2020; Wu et al., 2020). The viral proteases 3CL^pro^ and PL^pro^ are the key proteins for the proteolytic processing of the polyproteins that are helpful for initiating replication, whereas RdRp is a vital replication machinery of the virus, making multiple copies of the RNA genome. Therefore, these proteins have been considered as promising drug targets in the treatment of viral diseases (Fehr and Perlman, 2015; Yadav et al., 2020). Recently, several drugs including Lopinavir, Ritonavir, Remdesivir, and Favipiravir were repurposed and are currently under clinical trials to assess their anti-viral efficacy and safety for the treatment of COVID-19 (Drożdżal et al., 2020). Similarly, the recent study reviewed various natural compounds such as Artemisinin, Berbrine, Cholcgicine, Glycyrrhizin, Hanfangchin A, Lactoferrin, Quercetin, Resveratrol Vitamin C, and Vitamin D were found effective against SARS-CoV-2 druggable targets and are currently in clinical trials (Romeo et al., 2021). The previously mentioned study confirms that natural compounds can act as lead scaffolds for drug design or help in improving immunity. Islam et al., also reviewed the potential of natural products and their derivatives such as lycorine, homoharringtonine, silvestrol, ouabain, tylophorine, and 7-methoxycryptopleurine which were reported to inhibit SARS-CoV and its variant key proteins in nanomolar concentrations (Islam et al., 2020). Unfortunately, to date, none of the drugs have been identified as specific inhibitors for COVID-19 viral infection and research is ongoing to explore novel specific compounds. Correspondingly, the emerging source of natural products that have not been explored against COVID-19, is the marine habitat, home to more than 20,000 natural products discovered thus far (Blunt et al., 2016). It has been reported that marine compounds have antimicrobial, antiviral, antidiabetic, anticancer, and anti- inflammatory properties (Mayer et al., 2010, 2011; Pangestuti and Kim, 2017). Intriguingly, coumarin derived compounds from marine sponge *Axinella* sp. and phlorotannins isolated from marine algae *Eckolina cava* were able to target SARS 3CL^pro^ through *in vitro* studies (De Lira et al., 2007; Park et al., 2013). A molecular docking-based study recently reported the antiviral potential of marine-derived compounds including oleic acid, saringosterol, β-Sitosterol, Caulerpin, Glycoglycerolipids, Kjellmanianone, and Loliolide isolated from red, green, and brown macroalgae as candidate inhibitors against 3CL^pro^, the Spike protein, and the ACE-2 receptor of SARS-CoV-2 (El-Mageed et al., 2021). Another study also reviewed the therapeutic role of *Spirulina* algae-derived nutraceuticals in boosting the adaptive and innate immunity against coronavirus as well as other viral diseases (Ratha et al., 2021). Similarly, Ray et al. (2020) reviewed the potential role of marine-derived bioactive polysaccharides including fucan sulfates, ulvan, alginate, agarans, carrageenans, and galactans from red seaweeds against diverse. Correspondingly, the authors have reported on the key role of phlorotannins derived from *Sargassum spinuligerum* brown algae for COVID-19 therapeutics (Ray et al., 2020). We therefore designed our study to target viral key replication proteins using marine derived natural compounds, by using a series of computational methods. A drug-like database was prepared employing Lipinski’s rule of five and ADMET descriptors from a MNP library of 14,492 compounds. A total of 2,033 compounds were retrieved. Molecular docking based virtual screening was performed using GOLD software for selected proteins. Before the docking experiments, the docking method was validated with co- crystallized ligands (REF) and an acceptable binding pose with lower RMSD was selected (Supplementary Figure 1). The docking results confirm the identification of five, four, and five compounds binding to 3CL^pro^, PL^pro^, and RdRp, respectively, on the basis of a cluster analysis, higher docking scores than the reference compounds (Supplementary Table 1), and key residue interactions. Since molecular docking does not involve physiological conditions and real-time behavior of the protein-ligand interactions, selected compounds were subjected to MD simulations to check their stability and binding affinity towards respective target proteins (Blunt et al., 2016; Mathpal et al., 2020).

The structural analysis of the SARS-CoV-2 3CL^pro^ crystal structure bound with α-ketoamide revealed the inhibitor targets of His41, Phe140, Gly143, Cys145, His164, and Glu166 through hydrogen bonds. Furthermore, it has been elucidated that Glu166 is indispensable in maintaining the shape of the catalytic pocket and also for maintaining the enzyme conformation in an active state (Zhang et al., 2020). In a drug repurposing study, interaction energy of Glu166 with hit compounds was reported to be significantly low, thus confirming its imperative role in drug interaction (Wang, 2020). Recently published studies targeting 3CL^pro^ revealed that hydrogen bonds with catalytic residues His41 and Cys145 is essential for the inhibition of 3CL^pro^ (Alexpandi et al., 2020; Iftikhar et al., 2020; Kumar et al., 2020; Tahir ul Qamar et al., 2020; Vardhan and Sahoo, 2020; Zhang et al., 2020). In the present study, it was observed that our hits occupied a catalytic site similar to the REF compound. As explained earlier, the interaction with catalytic triad His41, Cys145, and Glu166 is significant for the inhibition of 3CL^pro^. Interestingly, it was noticed that our hits formed hydrogen bonds with either His41 or Glu166 or both (Figure 4). Additionally, Hit1 formed stable hydrogen bonds with other active site residues Val186, Thr190, and Gln192, whereas Hit2 formed a hydrogen bond with Gln192. In a recent repurposing study on COVID-19, residues His41, Met49, Asn142, His164, Met165, Glu166, and Gln189 were reported to be common hotspots for inhibiting 3CL^pro^ (Wang, 2020). Likewise, inspection for these residues revealed that Hit1 targeted hotspot residues His41, Met49, Cys145, Met165, and Gln189, while Hit2 targeted residues His41, Met49, Cys145, His164, Met165, Glu166, and Gln189 characterized by various hydrogen, hydrophobic, and π-alkyl bonds (Figure 4 and Supplementary Table 2). Similarly, a previously established binding mode for Lopinavir and Ritonavir against 3CL^pro^ was obtained during our analysis which provides support for the binding mode of identified Hit compounds (Bolcato et al., 2020; Kumar et al., 2021). Recently, a terpenoid (T3) from marine sponge *Cacospongia mycofijiensis* was also identified through *in silico* studies against SARS CoV-2 3CL^pro^ showing similar binding interactions like our hit molecules (Sepay et al., 2021). A recent pharmacophore-based study was published where authors screened the same MNP library against SARS-CoV-2 3CL^pro^ (Gentile et al., 2020) and identified 17 different hits with different scaffolds. It is worth noting that their identified hits are different from our proposed ones (Gentile et al., 2020). The BFE calculations also revealed significantly better binding energies for our hits with Hit1 demonstrating a BFE of -104.37 kJ/mol and Hit2 with a BFE of -102.90 kJ/mol than REF compound α-ketoamide with a BFE of -96.64 kJ/mol (Table 1). Additionally, BFE values for Lopinavir and Ritonavir was found to be -101.13 and -97.40 kJ/mol, respectively. It was observed that both drugs have better binding affinity than the co-crystallized REF inhibitor but interestingly, our Hit compounds displayed slightly better affinity than both the drugs (Table 1). The above overall analyses clearly indicates that our hits have better binding affinity than α-ketoamide against 3CL^pro^. Another SARS-CoV-2 protease PL^pro^ performs the hydrolysis of the peptide bond on the carboxyl side of glycine residue which then results in the release of nsp1, nsp2, and nsp3 proteins essential for virus replication. The peptide inhibitor VIR251 bound crystal structure of PL^pro^ was solved recently and revealed that the inhibitor occupies the active site of protease and makes numerous interactions that are important for inhibition (Rut et al., 2020). Several studies involved in the inhibition of SARS-CoV-2 PL^pro^ published recently reveal that Cys111, Leu162, Asp164, Met208, Pro247, Pro248, Tyr264, and Tyr268 are the crucial residues for its inhibition (Amin et al., 2020; Arya et al., 2020; Naidoo et al., 2020; Rut et al., 2020; Sen Gupta et al., 2020; Shin et al., 2020). Our identified Hit1 and Hit2 was found to target key residues Asp164 and Tyr268 through the hydrogen bond, while Hit2 also formed additional hydrogen bonds with Arg166 (Figure 5). An interesting finding by Shin et al. (2020) reported that when a mutant of Tyr268 is treated with inhibitor GRL-0617, its activity reduces significantly, thus revealing the role of Tyr268 in PL^pro^ inhibition. Our identified hits were observed to bind with Tyr268 characterized by hydrogen bonds. This analysis revealed that our hits can inhibit PL^pro^ in a similar way as GRL-0617. The similar residues also targeted by the REF drug Ritonavir provides support for Hit compound binding behavior (Supplementary Figure 4). Moreover, our hits targeted additional residues essential for inhibition, like Cys111, Leu162, Met208, Pro247, Pro248, and Tyr264 as stated earlier, via π-alkyl and van der Waals interactions (Figure 5 and Supplementary Table 2). BFE calculations for PL^pro^ revealed substantial differences in BFE values for identified Hits and REF inhibitors. The Hit1 and Hit 2 displayed a BFE value of -92.57 kJ/mol and -82.17 kJ/mol, whereas the BFE value for the REF peptide inhibitor VIR251, Lopinavir, and Ritonavir was observed as -51.82, -60.84, and -70.74 kJ/mol respectively (Table 1). It is worth noting that Lopinavir and Ritonavir displayed better binding affinity towards PL^pro^ than the co-crystalized ligand but not better than identified Hit compounds. The overall analysis provides adequate support to propose our hits as potential leads to be targeted against PL^pro^.

**FIGURE 6:**
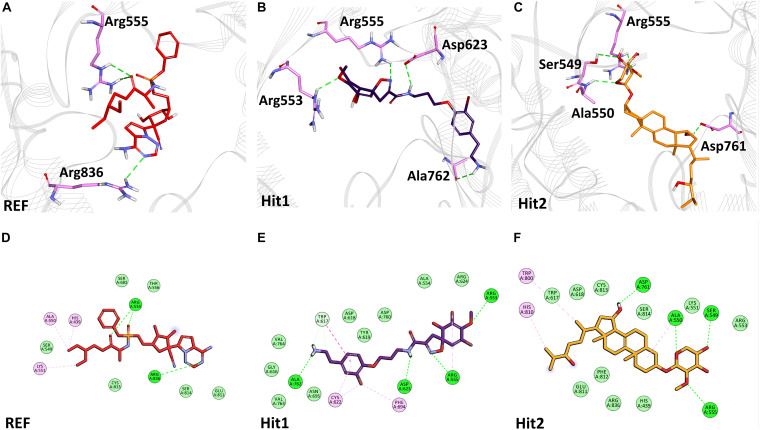
Binding mode of interaction in 3D **(upper panel)** for hits with the active site of SARS-CoV-2 RdRp, **(A)** REF inhibitor, **(B)** Hit1 and **(C)** Hit2. The protein in background is shown as wire representation with gray color, interacting residues are displayed as pink sticks while REF, Hit1 and Hit2 are displayed as red, purple and orange color sticks, respectively. The lower panel displaying 2D interactions, **(D)** REF, **(E)** Hit1 and **(F)** Hit2 with active site interacting residues of SARS-CoV-2 RdRp. The hydrogen bonds are shown as green dashed lines, while the π-alkyl and van der Waals interactions are displayed as pink and light green spheres, respectively.

The RdRp, which is a key component of the replication machinery of the virus to make multiple copies of the RNA genome, is a potential therapeutic target (Jiang et al., 2020). The cryo-EM structure of RdRp nsp12 in complex with nsp7 and nsp8 has been recently solved, revealing that RdRp consists of three domains- fingers, palm, and thumb (Gao et al., 2020). Recently, it has been shown that Remdesivir can bind in the active site of RdRp which is mainly in the palm domain (Yin et al., 2020). A literature survey further revealed Ala550, Arg553, Arg555, Asp618, Ser759, Asp760, and Asp761 of SARS-CoV-2 RdRp to be vital residues for its inhibition (Alexpandi et al., 2020; Barage et al., 2020; Elfiky, 2020; Gao et al., 2020; Iftikhar et al., 2020; Koulgi et al., 2020; Singh et al., 2020; Vardhan and Sahoo, 2020; Yin et al., 2020). Our identified Hit1 formed hydrogen bonds with key residues Arg553 and Arg555 and additionally targeted other catalytic residues Asp623 and Ala762. Hit2 was also observed to bind with Ala550 and Arg555 along with residues Ser549 and Asp761 *via* hydrogen bonds essential for inhibition of RdRp. More importantly, Remdesivir was perceived to form a smaller number of interactions than our identified hits (Figure 6). Similar interactions of Remdesivir with RdRp was shown recently through MD simulations thus providing support for our findings (Koulgi et al., 2020). Additionally, our hits displayed numerous interactions with residues Asp618, Cys622, Phe694, Trp800, His810, Cys813, Asp760, Ser814, and Arg836 characterized by π-alkyl and van der Waals interactions (Figure 6 and Supplementary Table 2). Similarly, BFE of identified RdRp hits through MM/PBSA was calculated with a Hit1 BFE value of -111.52 kJ/mol, followed by a Hit2 BFE value of -63.12 kJ/mol. The identified RdRp hits displayed a significantly better binding energy than the Remdesivir BFE value of -55.50 kJ/mol (Table 1). In addition to the above analysis, identified hits were also scrutinized for their pharmacokinetic properties such as intestinal absorption, CYP P450 inhibition, and their hepatoxicity, by an online platform pkCSM and were compared with reference compounds. The *in silico* PK results demonstrated that identified MNPs are non-mutagenic, are easily absorbed in the human intestine, display Caco-2 permeability, do not inhibit CYP enzymes, and are essentially non-toxic (Table 2). Overall, we believe that these MNP hits may be efficient and effective drug candidates as novel therapeutics against COVID-19 and can be recommended for further *in vitro* studies.

## Conclusion

In this study, marine derived drug-like natural products were used for the inhibition of SARS-CoV-2 replication proteins 3CL^pro^, PL^pro^, and RdRp. A drug-like database was generated by applying Lipinski’s rule of five and ADMET descriptors on a database of 14,492 compounds generating a total of 2,033 molecules. Detailed docking analysis revealed that 14 compounds were able to display desirable interactions with the protein active site. Selected compounds were then subjected to MD simulations to check their stability and binding affinity against respective target proteins, supplemented with a rigorous MM/PBSA analysis. Six marine compounds displayed better molecular characteristics and binding free energies than the reference inhibitors, characterizing them as potential leads. Detailed analyses of *in silico* pharmacokinetic properties of the six identified hits demonstrated exceptional properties indicative of their non-toxic and non-mutagenic nature. We anticipate that our identified hits could substantially influence the development of novel therapeutics against COVID-19 in the future.

## Data Availability Statement

The original contributions presented in the study are included in the article/Supplementary Material, further inquiries can be directed to the corresponding author.

## Author Contributions

VK, SP, and KL conceived the idea of the project and analyzed the results. VK and SP performed the experiments and compiled the manuscript. All the authors have read and approved the manuscript for submission.

## Conflict of Interest

The authors declare that the research was conducted in the absence of any commercial or financial relationships that could be construed as a potential conflict of interest.

## Footnotes

1 http://docking.umh.es/downloaddb

2 www.rcsb.org

3 http://biosig.unimelb.edu.au/pkcsm/

4 https://pubchem.ncbi.nlm.nih.gov/

## Supplementary Material

Supplementary Figure 1 Validation of docking parameters using co-crystallized ligands (red) and docked pose of **(A)** 3CL^pro^ (cyan), **(B)** PL^pro^ (green), and **(C)** RdRp (pink).

Supplementary Figure 2 MD simulation analyses displaying backbone RMSD **(A)** 3CL^pro^, **(B)** PL^pro^ and MM/PBSA binding free energy analyses **(C)** 3CL^pro^, **(D)** PL^pro^ for Lopinavir and Ritonavir.

Supplementary Figure 3 Binding mode of interaction in 3D **(upper panel)** and 2D **(lower panel)** for Lopinavir and Ritonavir with the active site of SARS-CoV-2 3CL^pro^. The protein in background is shown as wire representation with gray color, interacting residues are displayed as cyan sticks while Lopinavir and Ritonavir are displayed as black and brown color sticks, respectively. The hydrogen bonds are shown as green dashed lines, while the π-alkyl and van der Waals interactions are displayed as pink and light green spheres, respectively.

Supplementary Figure 4 Binding mode of interaction in 3D **(upper panel)** and 2D **(lower panel)** for Lopinavir and Ritonavir with the active site of SARS-CoV-2 PL^pro^. The protein in background is shown as wire representation with gray color, interacting residues are displayed as cyan sticks while Lopinavir and Ritonavir are displayed as black and brown color sticks, respectively. The hydrogen bonds are shown as green dashed lines, while the π-alkyl and van der Waals interactions are displayed as pink and light green spheres, respectively.

Supplementary Table 1 Molecular structures and docking scores of drug-like and reference compounds.

Supplementary Table 2 Intermolecular interactions formed by identified hits with 3CL^pro^, PL^pro^, and RdRp.
