# Supplementary material for: Computational Simulations Identified Marine-Derived Natural Bioactive Compounds as Replication Inhibitors of SARS-CoV-2
Source: Front Microbiol. 2021 Apr 21;12:647295. doi: 10.3389/fmicb.2021.647295 (PMC8097174; doi:10.3389/fmicb.2021.647295)
Supplement: Supplementary Figure 1 — Validation of docking parameters using co-crystallized ligands (red) and docked pose of (A) 3CLpro (cyan), (B) PLpro (green), and (C) RdRp (pink). [file Data_Sheet_1.docx]

Supplementary Material

Computational Simulations Identified Marine-Derived Natural Bioactive Compounds as Replication Inhibitors of SARS-CoV-2

Vikas Kumar^1†^, Shraddha Parate^2†^, Sanghwa Yoon^1^, Gihwan Lee^2^ and Keun Woo Lee^1*^

^1^Division of Life Sciences, Department of Bio & Medical Big Data (BK4 Program), Research Institute of Natural Science (RINS), Gyeongsang National University (GNU), 501 Jinju-daero, Jinju 52828, Republic of Korea

^2^ Division of Applied Life Science, Plant Molecular Biology and Biotechnology Research Center (PMBBRC), Gyeongsang National University (GNU), 501 Jinju-daero, Jinju 52828, Republic of Korea

*** Correspondence:**Keun Woo Lee
[kwlee@gnu.ac.kr](mailto:kwlee@gnu.ac.kr)

†These authors contributed equally to this work.

# Supplementary Tables

Supplementary Table 1. Molecular structures and docking scores of drug-like and reference compounds.

| **Compound**  **Name** | **Molecular Structure** | **Docking Score** | |
| --- | --- | --- | --- |
|  |  | **Goldscore** | **Chemscore** |
| **SARS-CoV-2 3CL^pro^** | | | |
| **Compound1** |  | 67.01 | -23.86 |
| **Compound2** |  | 66.07 | -23.69 |
| **Compound3** |  | 64.40 | -29.37 |
| **Compound4** |  | 62.85 | -28.33 |
| **Compound5** |  | 61.58 | -21.70 |
| **REF** |  | 61.23 | -17.76 |
| **SARS-CoV-2 PL^pro^** | | | |
| **Compound 1** |  | 65.97 | -31.82 |
| **Compound 2** |  | 65.58 | -33.24 |
| **Compound 3** |  | 60.34 | -32.20 |
| **Compound 4** |  | 57.77 | -30.22 |
| **REF** |  | 56.09 | -23.41 |
| **SARS-CoV-2 RDRP** | | | |
| **Compound 1** |  | 72.11 | -24.27 |
| **Compound 2** |  | 69.31 | -22.65 |
| **Compound 3** |  | 68.37 | -25.65 |
| **Compound 4** |  | 67.74 | -26.42 |
| **Compound 5** |  | 66.81 | -22.63 |
| **REF** |  | 60.95 | -22.41 |

**Supplementary Table 2.** Intermolecular interactions formed by identified hits with 3CL^pro^, PL^pro^ and RdRp.

| Complex Name | | Hydrogen Bond Interaction | | | | van der Waals Interactions | π -π /π-alkyl interactions |
| --- | --- | --- | --- | --- | --- | --- | --- |
|  |  | Amino acid | Amino acid atom | Ligand atom | Distance (<3.0 Å) |  |  |
| 3CL^pro^ | Hit1 | His41 | HE2 | O1 | 2.02 | Thr25, Val42, Cys145, Leu167, Pro168, Phe185, Arg188, Gln189, Ala193 | Met49, Met165 |
|  |  | Val186 | HN | O31 | 2.03 |  |  |
|  |  | Gln192 | O | H65 | 1.98 |  |  |
|  |  | Thr190 | HN | O22 | 2.78 |  |  |
|  | Hit2 | His41 | HE2 | O25 | 1.93 | Thr25, Thr26, Leu27, Gly143, His164, Met165, Leu167, Pro168, Asp187, Arg188, Gln189, Thr190 | Met49, Cys145 |
|  |  | Glu166 | O | H29 | 1.76 |  |  |
|  | REF | His41 | HE2 | O33 | 1.88 | Leu27, Tyr54, Asn142, Gly143, Cys145, Asp187, Arg188, Thr190 | Met49, Met165, Ala191 |
|  |  | Glu166 | HN | O28 | 1.82 |  |  |
|  |  | Gln189 | HE21 | O11 | 2.06 |  |  |
| PL^pro^ | Hit1 | Asp164 | OD2 | H41 | 1.77 | Asn109, Tyr112, Leu162, Gly163, Met208, Ala246, Ser245, Pro247, Pro248, Tyr268, Gln269, Gly271, Tyr273, Thr301, Asp302 | Met49, Met165, Ala191 |
|  |  | Tyr268 | O | H41 | 1.66 |  |  |
|  | Hit2 | Asp164 | OD2 | H39 | 2.72 | Asn109, Leu162, Gly163, Met208, Pro247, Gln269, Gly271, Tyr273, Thr301 | Pro248, Tyr264, Cys270 |
|  |  | Arg166 | HH21 | O1 | 2.65 |  |  |
|  |  | Tyr268 | O | H41 | 3.08 |  |  |
|  | REF | Gly163 | HN | O46 | 1.88 | Trp106. Asn109, Cys111, Tyr112, Leu162, Asp164, Met208, Ala246, Pro247, Pro248, Tyr268, Gln269, Cys270, His272, Tyr273 | Arg166 |
|  |  | Tyr264 | HH | O34 | 1.75 |  |  |
|  |  | Gly271 | O | H58 | 1.73 |  |  |
|  |  | Thr301 | HG1 | O18 | 1.94 |  |  |
|  |  | Asp302 | OD1 | H30 | 1.69 |  |  |
| RDRP | Hit1 | Arg553 | HE | O5 | 2.46 | Ala554, Gly616, Trp617, Asp618, Tyr619, Arg624, Asn695, Asp760, Val763, Val764 | Cys622, Phe694 |
|  |  | Arg555 | HH12 | N14 | 2.61 |  |  |
|  |  | Asp623 | OD2 | H41 | 2.68 |  |  |
|  |  | Ala762 | O | H55 | 2.57 |  |  |
|  | Hit2 | Ser549 | HG | O10 | 3.05 | His439, Lys551, Arg553, Trp617, Asp618, Glu811, Phe812, Cys813, Ser814, Arg836 | Trp800, His810 |
|  |  | Ala550 | HN | O1 | 2.53 |  |  |
|  |  | Arg555 | HE | O8 | 2.97 |  |  |
|  |  | Asp761 | OD2 | H75 | 2.13 |  |  |
|  | REF | Arg555 | HH21 | O9 | 2.89 | Ser549, Thr556, Ser682, Glu811, Cys813, Ser814 | His439, Ala550, Lys551 |
|  |  | Arg836 | HH11 | N14 | 2.94 |  |  |


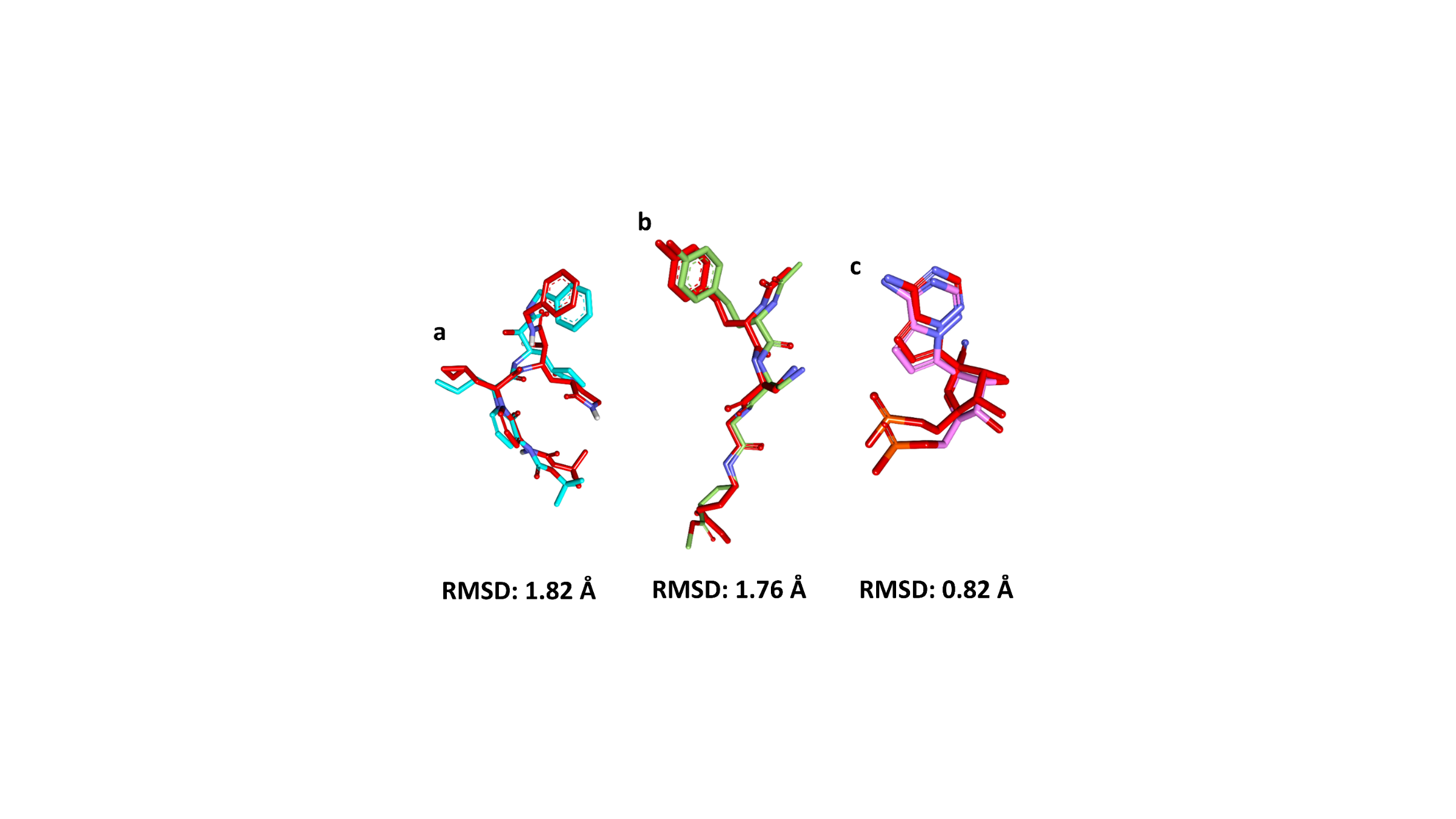


**Supplementary Figure 1.** Validation of docking parameters using co-crystallized ligands (red) and docked pose of a) 3CL^pro^ (cyan), b) PL^pro^ (green) and c) RdRp (pink).


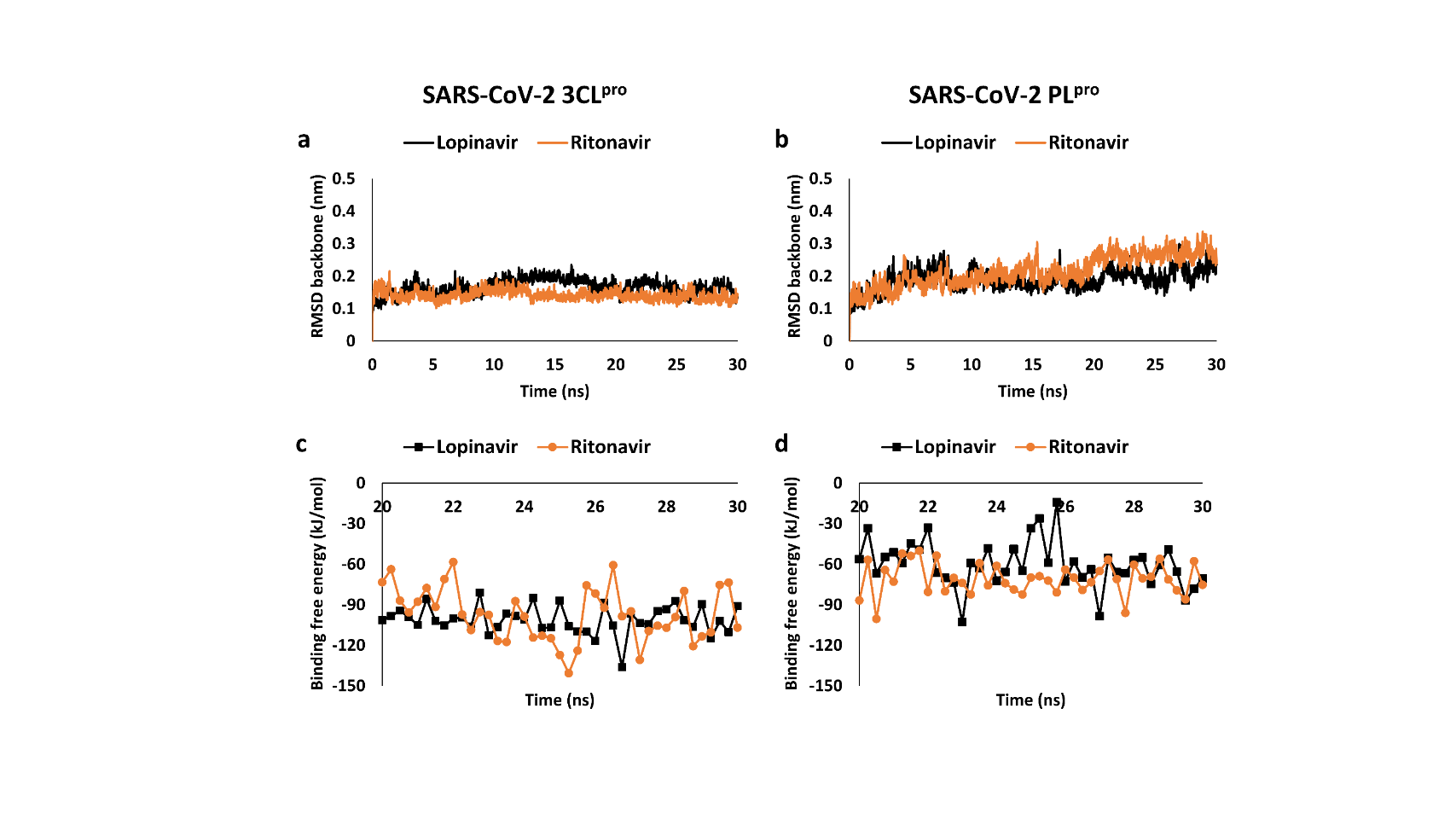


**Supplementary Figure 2.** MD simulation analyses displaying backbone RMSD a) 3CL^pro^, b) PL^pro^ and MM/PBSA binding free energy analyses c) 3CL^pro^, d) PL^pro^ for Lopinavir and Ritonavir.

**
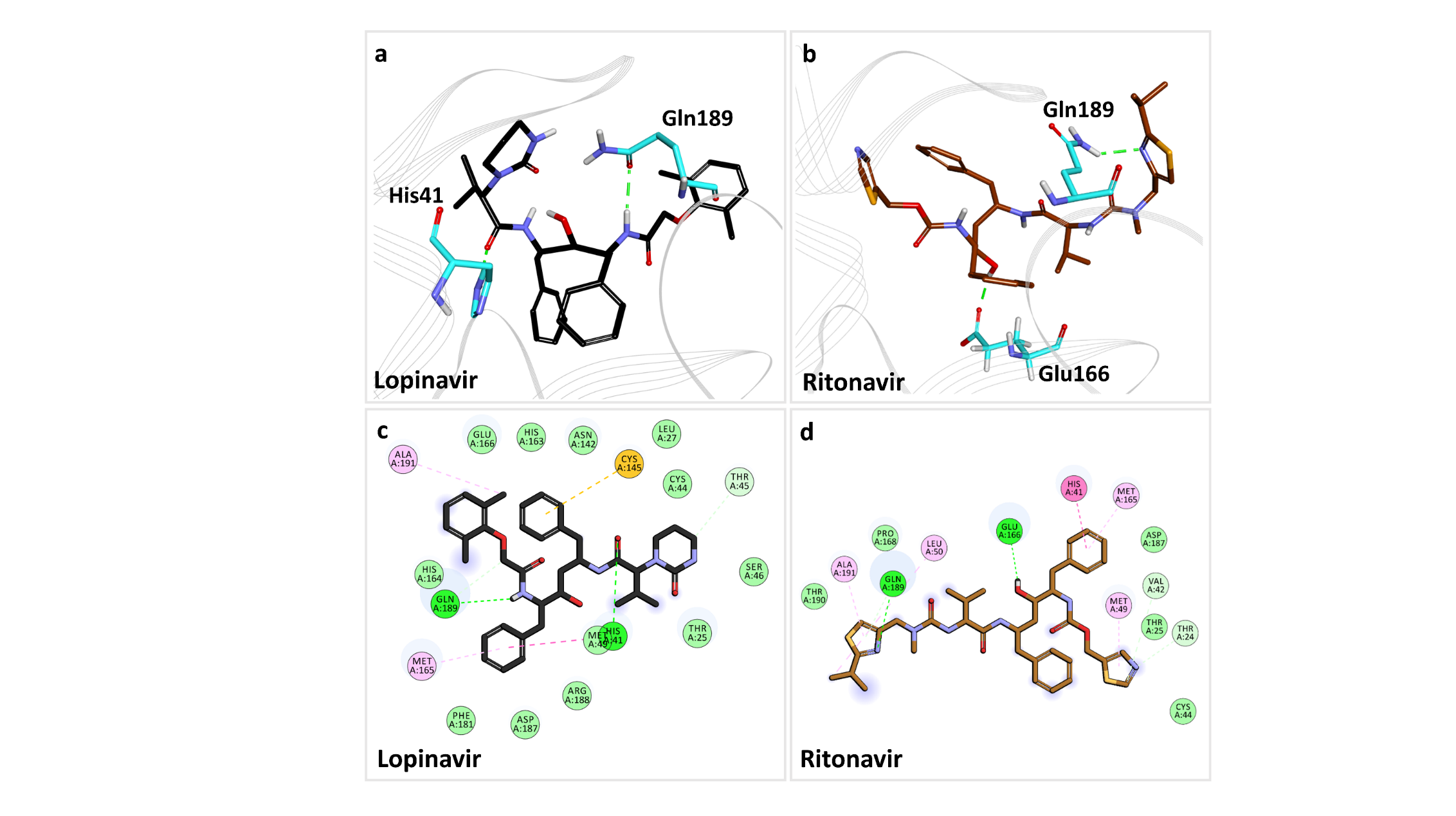
**

**Supplementary Figure 3.** Binding mode of interaction in 3D (upper panel) and 2D (lower panel) for Lopinavir and Ritonavir with the active site of SARS-CoV-2 3CL^pro^. The protein in background is shown as wire representation with grey color, interacting residues are displayed as cyan sticks while Lopinavir and Ritonavir are displayed as black and brown color sticks, respectively. The hydrogen bonds are shown as green dashed lines, while the π-alkyl and van der Waals interactions are displayed as pink and light green spheres, respectively.

**
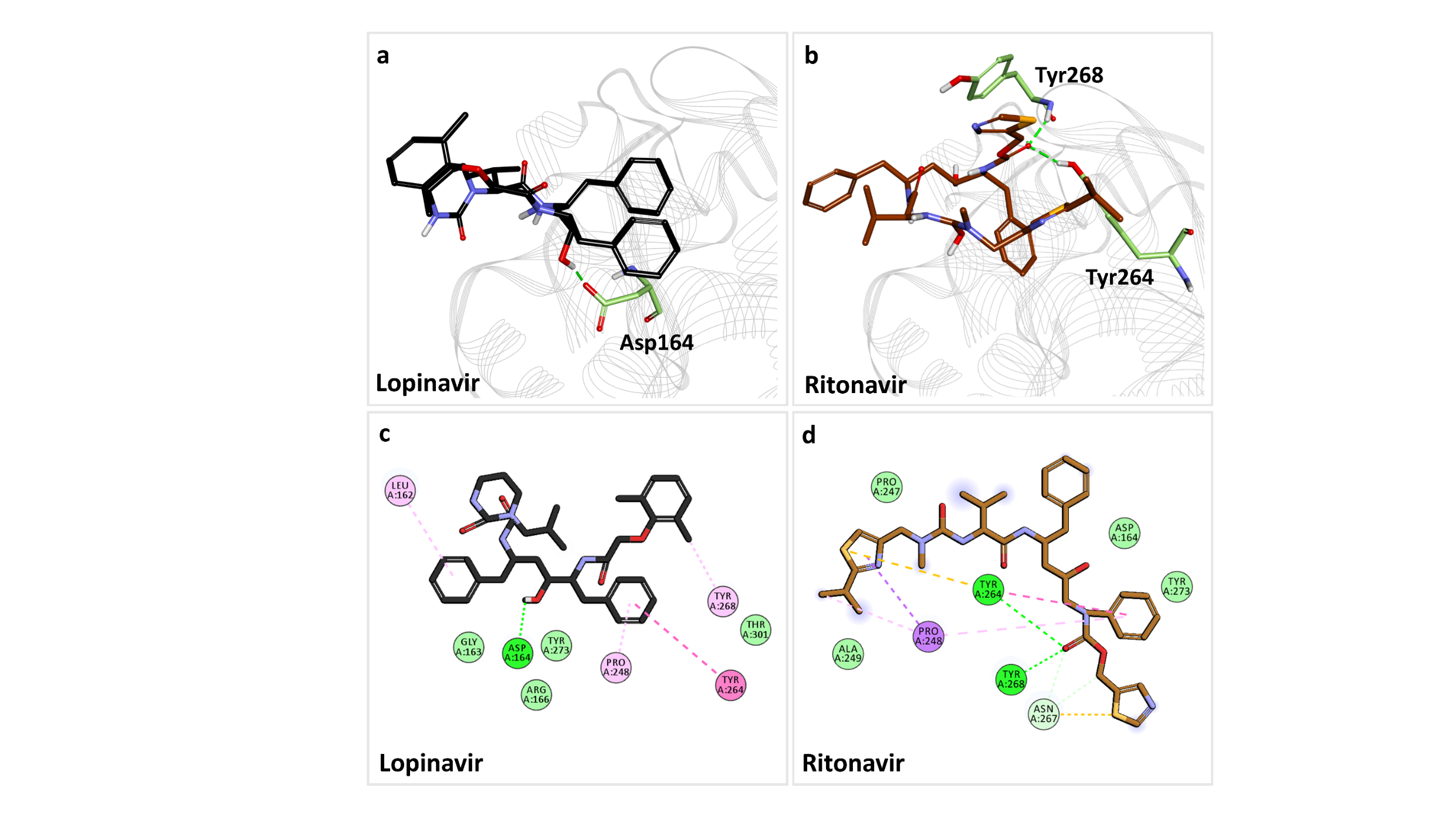
Supplementary Figure 4.** Binding mode of interaction in 3D (upper panel) and 2D (lower panel) for Lopinavir and Ritonavir with the active site of SARS-CoV-2 PL^pro^. The protein in background is shown as wire representation with grey color, interacting residues are displayed as cyan sticks while Lopinavir and Ritonavir are displayed as black and brown color sticks, respectively. The hydrogen bonds are shown as green dashed lines, while the π-alkyl and van der Waals interactions are displayed as pink and light green spheres, respectively.
